# Phytochemical Omics in Medicinal Plants

**DOI:** 10.3390/biom10060936

**Published:** 2020-06-21

**Authors:** Jen-Tsung Chen

**Affiliations:** Department of Life Sciences, National University of Kaohsiung, Kaohsiung 811, Taiwan; jentsung@nuk.edu.tw

Medicinal plants are used to treat diseases and provide health benefits, and their applications are increasing around the world. A huge array of phytochemicals have been identified from medicinal plants, belonging to carotenoids, flavonoids, lignans, and phenolic acids, and so on, having a wide range of biological activities. In order to explore our knowledge of phytochemicals with the assistance of modern molecular tools and high-throughput technologies, this special issue collects recent innovative original research and reviews. As summarized below, 27 papers are published in this special issue, which can be divided into four subtopics, as described below.

## 1. Mechanistic Insights into Bioactivities

### 1.1. Anti-Inflammation

Inflammation undergoes physiological and pathological responses caused by diverse stimuli such as microbial infections, injury, and traumata. Zwirchmayr et al. identified anti-inflammatory compounds that exhibit nuclear factor kappa-light-chain-enhancer of activated B cells (NF-κB) inhibiting ability from the extract of masterwort (*Peucedanum ostruthium* (L.) Koch) through a holistic omics-based tool, namely Eliciting Nature’s Activities (ELINA) [1]. They successfully isolated four furanocoumarins, one coumarin, and one chromone. According to the results presented, they suggest that ELINA is practicable and may effectively accelerate the process of natural product-based drug discovery in the future.

Inflammation is part of immunity, Yeh and Lin investigated the immunomodulatory potential of steam-distilled essential oils (SDEOs) from *Acorus gramineusand* and *Euodia ruticarpa* cultivated in Taiwan [2]. They found SDEOs to be rich in flavonoids, polyphenols, and saponins, and additionally, SDEOs, particularly from *Euodia ruticarpa*, possess immunomodulatory ability by shifting the balance of type 1 and type 2 T helper cells in primary splenocytes and inhibiting inflammation in peritoneal macrophages in vitro.

### 1.2. Anti-Oxidative Stress

Oxidative stress is one of the main processes in human disorders. Wang et al. contributed a review on bioactive compounds for treating oxidative stress-related human disorders involving the toxic reactive aldehyde 4-hydroxynonenal (4-HNE), an advanced lipid peroxidation end product [3]. A number of related disorders are listed, such as cardiovascular injury, eye damage, liver injury, neurotoxicity, neurological disorder and energy metabolism disorder in order to discuss their prevention and treatment by bioactive compounds from particular medicinal plants, and finally, possible strategies for future research and applications combating deleterious effects induced by 4-HNE are proposed.

### 1.3. Bioinformatics

In research related to traditional herbal medicine (THM), network pharmacology (NP) is a useful tool for exploring the potential mechanisms of therapeutic effects that act on multiple targets. Lee et al. constructed the networks of co-author and affiliation to analyze the trend of methodologies utilized by researchers [4]. Firstly, they found a dramatic increase in THM-NP studies in the last ten years. Additionally, the Traditional Chinese Medicine Systems Pharmacology Database and Analysis Platform was the most frequently utilized, and the methodology for constructing a compound-target network had achieved the greatest progress.

Jeyasri et al. used a system pharmacology approach to develop a strategy for multi-target treatment based on traditional Ayurvedic medicine, *Bacopa monnieri*, for neurological diseases, focusing on Alzheimer’s disease (AD) and Spinocerebellar ataxia (SCA) [5]. When compared with commercially available drugs, it was revealed that the constituents of *Bacopa monnieri* have asiatic acid and loliolide for treating AD and SCA. In addition, various potential actions from the bioactive compounds were predicted, particularly benefiting cognitive function.

The extracts of some *Garcinia* species have an array of bioactivities in the treatment of adipogenesis, obesity, cardiovascular diseases, diabetes, inflammation, and cancer. Chen et al. reviewed the molecular docking approach for searching candidate agents for the treatment of diabetes using a number of critical hypoglycemic targets [6]. Altogether, some benzopyrans and triterpenes that existed in *G. linii* were proposed to be the chief components for regulating blood glucose.

## 2. Treatment of Diseases

### 2.1. Cancers

Due to the high morbidity and mortality of oral cancer, there is a demand to develop drugs for inhibiting local invasion and metastasis of cancer cells. It has been reported that Withaferin A (WFA), a steroidal lactone isolated from *Withania somnifera*, could inhibit the migration of cancer cells at high cytotoxic concentrations. Interestingly, Yu et al. further prove that WFA in a relatively low concentration of 0.5 µM inhibited oxidative stress-mediated migration as well as invasion in oral cancer Ca9-22 cells, and they suggest that it has the potential to inhibit metastasis in oral cancer therapy [7]. Velmurugan et al. use a cell-proliferation inhibitory flavonoid, luteolin-7-O-glucoside, to further test its effect on metastasis of oral cancer [8]. On the basis of their results, they propose that luteolin-7-O-glucoside inhibits cell migration and invasion by regulating the expression of matrix metalloproteinase-2 and extracellular signal-regulated kinase pathway.

Non-small-cell lung cancer (NSCLC) accounts for approximately 85% of all lung cancer and is the major cause of death by cancer in the world. Oh et al. investigated the anti-cancer potential of licochalcone D (LCD), a flavonoid isolated from *Glycyrrhiza inflata*, using an epidermal growth factor receptor (EGFR) mutant NSCLC cell line [9]. They found that LCD inhibits the activity of EGFR and hepatocyte growth factor receptor, induces reactive oxygen species-dependent apoptotic cell death, and inhibits the proliferation of cancer cells.

Nasopharyngeal carcinoma (NPC) has a unique geographical and ethnic distribution. Its high prevalence in Asia accounts for over 80% of new cases globally. Liu et al. studied the anticancer activity of asiatic acid (AA), extracted from *Centella asiatica*, using two cisplatin-resistant NPC cell lines [10]. They demonstrated that AA significantly reduced the cell viability in both cell lines through p38-α mitogen-activated protein kinase (MAPK)-mediated phosphorylation and activation of bcl-2-like protein 4 (Bax) in vitro.

Inflammatory bowel disease (IBD) is chronic intestinal and colorectal inflammation, which is highly capable of developing into colorectal cancer (CRC). Zheng et al. evaluated bioactivities of a clerodane diterpene, 16-hydroxycleroda-3,13-dien-15,16-olide (HCD) using an IBD mouse model and two human CRC cell lines [11]. It was demonstrated that the inflammatory symptoms of IBD mice could be ameliorated by HCD treatments, and additionally, in vitro tests confirmed that HCD-induced apoptosis may involve both extrinsic and intrinsic pathways.

Khan et al. reviewed the recent advances in the use of active phytochemicals (APs) against cancers by searching relevant keywords in reliable academic databases, selecting twenty medicinal plants to discuss in detail [12]. It was revealed that APs were effective for treating a number of cancer cell lines, and altogether, the inhibitory effects were mainly exerted by damaging DNA and activating enzymes that cause apoptosis. In addition, the anticancer activity of some APs was confirmed using in vivo animal models.

There are more than 1300 *ent*-kaurane diterpenoids that have been identified, most of which belong to the genus *Isodon*, and many possess anticancer potential. Sarwar et al. provide a review of the plant sources, biological targets and mechanistic pathways of *ent*-kaurane diterpenoids [13]. They indicate that the anticancer activities of such compounds are chiefly contributed by the regulation of apoptosis, autophagy, cell cycle arrest, and metastasis. The key regulators in each process were discussed in detail; for example, the most common metastatic target proteins are matrix metalloproteinases (MMP-2 and MMP-9), vascular endothelial growth factor (VEGF), and VEGF receptor.

### 2.2. Neurological Diseases

Quercetin, a flavonoid with an array of pharmacological effects, is widely available in fruits and vegetables. It has been demonstrated that quercetin possesses a neuron-protective effect by alleviating oxidative stress and inflammation. Khan et al. further refine the effect and underlying mechanisms of quercetin on AD, with an emphasis on cognitive performance [14]. Finally, quercetin is proposed to offer potential as a lead compound for clinical application in such disease.

Another flavonoid, naringenin, also has neuroprotective ability. Nouri et al. refined the current knowledge of pharmacological targets, signaling pathways, molecular mechanisms, and the clinical perspective of such compounds [15]. Additionally, systems for delivering naringenin were discussed.

### 2.3. Liver Diseases

Liver failure frequently leads to hepatic encephalopathy (HE), which has a spectrum of neuropsychiatric abnormalities. Baek et al. studied the effect of *Rheum undulatum* and *Glycyrrhiza uralensis* extract mixture (RG) on HE based on their anti-inflammatory and antioxidant properties [16]. Firstly, they identified seven bioactive ingredients in RG that had been predicted to be effective in treating neurological diseases. Then, they used a CCl_4_-induced HE mouse model to unravel the therapeutic mechanisms of RG. Based on their results, RG consistently relieved HE symptoms by preserving blood–brain barrier permeability, and thus offers considerable potential for treating chronic liver disease as well as HE.

### 2.4. Cardiovascular Diseases

Cardiovascular diseases (CVDs) are the leading cause of death in the world, and are mainly related to atherosclerosis. Toma et al. refined the recent knowledge regarding the effects and underlying molecular mechanisms of phenolic compounds on atherosclerosis, especially involving dyslipidemia, oxidative stress, and inflammation [17]. In this review, the authors suggest future research directions for alternative therapy of CVDs using phenolic compounds, and encourage the adequate consumption of foods containing natural phenolic compounds in order to prevent such diseases.

### 2.5. Fracture Healing

Green tea is a popular drink that benefits human health, including having positive effects that increase bone mineral density and bone volume, while also diminishing osteoporotic fractures. Based on previous reports, a bioactive compound of tea catechin, (-)-epigallocatechin-3-gallate (EGCG) could promote bone defect healing in the distal femur, and this may be partly through the effect of bone morphogenetic protein-2 (BMP-2). Lin et al. further studied the effect of EGCG on tibial fracture healing in rats, and they concluded that the local treatment of EGCG accelerated fracture healing at least partly via BMP-2 [18].

## 3. Profiling, Extraction and Identification

### 3.1. Profiling

Ultraviolet-B (UV-B) radiation could induce stress in plants, and consequently, promotes the production of secondary metabolites through elevated defense responses. It has been reported that of the two *Astragalus* varieties, *A. mongholicus* has the higher tolerance to UV-B. Liu et al. further profiled the metabolites, particularly the phenolic compounds of *A. mongholicus*, when subjected to UV-B radiation [19]. Overall, they found that UV-B radiation could induce a tissue-specific strong shift from carbon assimilation to carbon accumulation of phenolic metabolism in roots, and it was activated by the upregulation of some relevant genes.

Chinese sage (*Salvia miltiorrhiza*) has been used as medicine for thousands of years for the treatment of a number of disorders, particularly by benefiting blood circulation systems. In a previous report, the polysaccharide fraction (PSF) of a symbiont, *Trichoderma atroviride*, was found to have a positive effect on the accumulation of tanshinones in the hairy root culture. Peng et al. further profiled the proteomic responses of the symbiont relationship, and they proposed that the PSF-induced accumulation of tanshinones may contribute through defense-related signaling involving jasmonic acid, leading to leucine-rich repeat protein synthesis [20].

### 3.2. Extraction and Identification

*Flos Chrysanthemi indici* is a traditional herb that has a number of bioactivities, including the antibacterial, anti-inflammatory, and antioxidant properties. Jing et al. studied the effect of extraction method on the yield and bioactivities of essential oils [21]. They suggest that solvent-free microwave extraction and supercritical fluid extraction are both good methods with higher yield that could simultaneously retain the antimicrobial activity of essential oils.

Deep eutectic solvents (DESs) are homogeneous solutions of quaternary ammonium salts, which have advantages including easy preparation, ecological friendliness, and so on. Zeng et al. tested the performance of six DESs in combination with five macroporous resins on isolation and purification of flavonoids and 20-hydroxyecdysone from *Chenopodium quinoa* Willd by preparative high-performance liquid chromatography [22]. They found the best combination of DES (choline chloride/urea) and macroporous resin (D101). Subsequently, three flavonoids and 20-hydroxyecdysone were successfully purified, and their chemical structures were identified.

Luteolin is a flavonoid and possesses an array of biological effects on hypertension, inflammatory diseases, and cancer. Juszczak et al. reviewed the current chromatographic techniques for analyzing luteolin and some derivatives [23]. They discussed the separation conditions for determining such compounds, as well as the pros and cons of each method.

To solve the excess structural information in the isolation of metabolites from natural products by high-resolution mass spectrometry, Lee et al. applied the Global Natural Product Social Molecular Networking platform together with hierarchical clustering analysis on the selectively isolate of lignans from *Trachelospermum asiaticum* [24]. Using this combined tool, eventually, they efficiently identified five dibenzylbutylrolactone-type lignans that possess cytotoxic activities against cancer cells.

*Curcuma* species (Zingiberaceae) have long been used as traditional medicine, food flavor and cosmetics. Pintatum et al. identified bioactive compounds from the *Curcuma aromatica* rhizome, including curcumin, curcumenol, curdione, germacrone, and zederone, and found that curcumin has the highest anti-inflammatory ability [25]. Additionally, the crude extract of *C. aromatica* demonstrated the highest inhibitory response to NF-κB activity when compared to the other three *Curcuma* species.

## 4. Biotechnology

### 4.1. Gene Transfer

Black nightshade (*Solanum nigrum*) is regarded as an herb that possesses bioactive compounds, particularly those with anti-tumor activities. Chhon et al. used transgenic black nightshade that overexpressed *AtPAP1* (*Arabidopsis thaliana* production of anthocyanin pigment 1), to investigate the effect of transgene on anthocyanin biosynthesis [26]. They found that transgenic plants produced a higher content of anthocyanin when compared to the control, and thus suggested that it could be a suitable platform for further studying AtPAP1-induced anthocyanin accumulation in response to environmental stress.

### 4.2. Nanoparticles

Green synthesis of nanoparticles (NPs) is an emerging field that is regarded as “green chemistry” due to the use of biological reducing agents including plant sources. Abbasi et al. made zinc oxide NPs that conjugated with the leaf extract from *Geranium wallichianum* (GW), namely GW-ZnONPs [27]. They tested a number of bioactivities, as well as the biocompatibility of GW-ZnONPs, and finally confirmed that it has considerable potential in biological applications.

## 5. Conclusions and Perspectives

Phytochemical omics is clearly an interesting topic and has attracted intensive studies focusing on anticancer, anti-inflammation, immunoregulation, anti-oxidative stress, anti-diabetes, fracture healing, and treatments for neurological, liver, and cardiovascular diseases. Additionally, recent advances in profiling, extraction, identification, and biotechnology are included. The findings offered by the contributors to this special issue may greatly aid the progress of phytochemical research. In the future, I hope that with the rapid development of molecular tools and approaches, particularly in integrative multi-omics and genome editing technologies, scientists will be able to further unravel the efficacy of phytochemicals for benefiting human health.
